# High resolution imaging reveals heterogeneity in chromatin states between cells that is not inherited through cell division

**DOI:** 10.1186/s12860-016-0111-y

**Published:** 2016-09-08

**Authors:** David Dickerson, Marek Gierliński, Vijender Singh, Etsushi Kitamura, Graeme Ball, Tomoyuki U. Tanaka, Tom Owen-Hughes

**Affiliations:** 1 Centre for Gene Regulation and Expression, College of Life Sciences, University of Dundee, Dundee, DD1 5EH UK; 2 Wellcome Trust Building, University of Dundee, Dow Street, Dundee, DD1 5EH UK

**Keywords:** Chromatin structure, Fluorescence microscopy, Live cell imaging, Epigenetic inheritance

## Abstract

**Background:**

Genomes of eukaryotes exist as chromatin, and it is known that different chromatin states can influence gene regulation. Chromatin is not a static structure, but is known to be dynamic and vary between cells. In order to monitor the organisation of chromatin in live cells we have engineered fluorescent fusion proteins which recognize specific operator sequences to tag pairs of syntenic gene loci. The separation of these loci was then tracked in three dimensions over time using fluorescence microscopy.

**Results:**

We established a work flow for measuring the distance between two fluorescently tagged, syntenic gene loci with a mean measurement error of 63 nm. In general, physical separation was observed to increase with increasing genomic separations. However, the extent to which chromatin is compressed varies for different genomic regions. No correlation was observed between compaction and the distribution of chromatin markers from genomic datasets or with contacts identified using capture based approaches. Variation in spatial separation was also observed within cells over time and between cells. Differences in the conformation of individual loci can persist for minutes in individual cells. Separation of reporter loci was found to be similar in related and unrelated daughter cell pairs.

**Conclusions:**

The directly observed physical separation of reporter loci in live cells is highly dynamic both over time and from cell to cell. However, consistent differences in separation are observed over some chromosomal regions that do not correlate with factors known to influence chromatin states. We conclude that as yet unidentified parameters influence chromatin configuration. We also find that while heterogeneity in chromatin states can be maintained for minutes between cells, it is not inherited through cell division. This may contribute to cell-to-cell transcriptional heterogeneity.

**Electronic supplementary material:**

The online version of this article (doi:10.1186/s12860-016-0111-y) contains supplementary material, which is available to authorized users.

## Background

Chromatin is a DNA-protein complex which provides cells with a framework for important packaging and regulatory functions. Biochemical reconstitution has provided profound insight into the structure of the nucleosome [1], the basic unit of chromatin organisation. Biophysical studies have also revealed that arrays of nucleosomes spontaneously reorganise to form chromatin fibres with a diameter of approximately 30 nm under the appropriate ionic conditions [2–4]. Proposed nucleosome arrangements for such fibres include the 1-start solenoidal and 2-start supercoiled models, as well as combinations of the two and less ordered structures [5–7]. However, studies of native chromatin provide evidence for well organised 30 nm fibres in only a few specialised cases [2, 8, 9]. Growing evidence from close-to-native-state methodologies favours the existence of relatively disordered arrays of nucleosomes in both mitotic [10–13] and interphase chromosomes [8, 12–15].

On a larger scale, studies in a variety of organisms have indicated that chromosomes are arranged into chromosomal territories [16–19]. These territories have been characterised as associations of megabase-scale topologically associated domains (TADs), which are thought to result from complex physical interactions between various regions of genomes [20–26] and this concept has been supported by Chromosome Conformation Capture strategies such as Hi-C and 5C [27]. Hi-C based approaches provide important insights into chromosome organisation, but many are subject to complications arising from a reliance on cross-linking as well as the difficulty in generating temporal information regarding the chromosomal interactions.

Chromatin organisation and mobility has also been studied in vivo using fluorescent tagging of genomic loci and analysing the cells via microscopy [14, 28–36]. These approaches make feasible the measurement of native chromatin characteristics such as compaction ratio, flexibility, and diffusive behaviour. Previously, comparison of genomic and physical separation in fixed cells has shown that squared inter-probe distances are related to genomic separation [37, 38]. The extent of folding has been observed to vary in different regions of metazoan chromosomes and between cell types [39]. Changes in compaction have also been observed to occur during differentiation at some loci [40] but not at others [41].

A great deal of effort has gone into the development of polymer models to describe chromatin structure. Random Walk, or a Self-avoiding Walk models were initially used to describe non-looping chromatin fibres [42]. Most recently, the diffusive properties of fluorescently tagged loci have been observed to be consistent with a rouse-like polymer [14]. Fractal models explain some of the observed properties of chromatin with organisation that is self-similar at different scales [24, 43] however, this is not fully supported by Hi-C data [44]. More recently, models including looping and polymer melt geometries have gained prevalence [42, 45, 46]. Looping models account for data from sources including 3C technologies and fluorescence in situ hybridisation, which indicate a non-linear relationship between spatial and genomic distance [46–48]. Looping interactions have the potential to juxtapose important regulatory regions as appropriate over time. Polymer Melt models currently hold widespread support given that they chromatin is modelled as relatively disordered arrays of nucleosomes rather than folded fibres consistent with cryo-EM and small angle X-ray scattering of native chromatin [11, 49]. The Strings and Binders Switch model, which is largely based on 3C data is also attractive in that it accounts for looping while simultaneously predicting nucleosomal DNA to be the predominant fibre [50].

Improvements in optics, image acquisition electronics, and live imaging techniques, together with the ability to label specific loci using fluorescent fusion proteins [51] enable the study of the dynamic nature of chromatin organization in cells in three dimensions over time with greater precision than has been possible previously. In this study we introduce distinct fluorescent tags flanking a range of genomic regions and track the motion of the labelled reporters using an OMX Blaze microscope. We describe a work flow that enables 3D live cell tracking with a mean measurement error of 63 nm. We find that within individual yeast cells the separation of operator sequences exhibits substantial variation over time. Genomic loci are able to reorganize extensively below a threshold of approximately 70 kb. However above this, there is a transition to independent motion constrained by the nuclear environment. Within a clonal population of cells the mean conformations of reporter loci vary significantly and can persist over time frames of 1–10 min. By comparing chromatin states in related mother-daughter cell pairs we observe no evidence for inheritance of chromatin conformation.

## Results

### A system to measure chromatin compaction in live cells

**Fig. 1:**
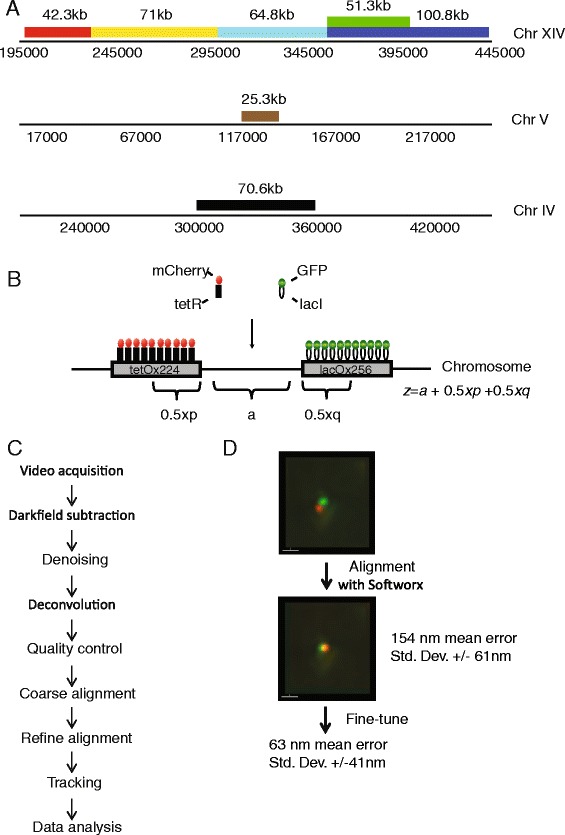
Establishment of a work-flow for live cell 4D imaging. **a** Seven sample strains were generated which introduced lac ×256 and tet ×224 operator arrays flanking regions on Chrs XIV, IV and V. **b** Operator arrays were detected using fluorescent tagged repressor proteins as indicated. A naming convention was adopted that includes the endogenous genomic distance (**a**) as well as half the distance of each operator array (0.5*x*_*p*_, 0.5*x*_*q*_). Not shown: An additional strain was generated with a single tetO ×224 array on Chr XIV which expressed both tetR-GFP and tetR-mCherry to produce colocalising green and red spots. This strain was used for channel alignment and mean measurement error estimation purposes. **c** Summary of the work flow for image processing. **d** Two stage channel alignment was found to improve standard error from 154 nm to 63 nm

In order to assay chromatin organization in vivo, we generated seven strains with fluorescently tagged chromosomal loci flanking various lengths of genomic DNA (Fig. 1a). The fluorescent repressor operator system (FROS) we adopted involves flanking different sides of reporter loci with arrays of 224 tet operators and 256 lac operators. These were then visualised though their interaction with mCherry TetR and GFP LacI (Fig. 1b). In order to mitigate the potential for arrays of repressor-bound operators to generate heterochromatin low concentrations of tetracycline were included in media to reduce tetR binding and a lacI point mutant was used [52].

As the fluorescent intensity of foci is likely to be centred at the midpoint of the lac or tet operator arrays, we adopted the naming convention of describing the genomic separation (z) present in these strains as *z* = *a* + 0.5*x*_*p*_ + 0.5*x*_*q*_ where *a* is the length of intragenic spacer and *x*_*p*_ and *x*_*q*_ are the lengths of the two flanking operators (Fig. 1b). Therefore, the strain with 60.6 kb of yeast genomic DNA flanked by lac and tet operator arrays was referred to as having 71 kb separation (60.6 kb genomic DNA + 10.4 kb of flanking operator array DNA), and so on. An additional strain was generated with colocalising green and red spots on Chr. XIV, for use as a control for channel alignment and measurement error.

Live cell 3-dimensional videos of these 8 strains were generated using the OMX microscope. The workflow used is summarised in Fig. 1c. Briefly, video acquisition was performed with CMOS cameras. To remove noise a dark field subtraction step was included as described in Materials and Methods. Subsequently, a second level of denoising was performed using ND-SAFIR using settings described by the Sedat Lab [53]. Deconvolution was performed using Softworx software. Quality control was performed as described in the Materials and Methods section. As the green and red channels are directed to different cameras on this system, channel alignment is critical to minimize translational, rotational, and scale errors. Initially we followed an established method which utilizes the imaging of multi-wavelength fluorescent beads to perform a coarse channel alignment. To improve the resolution that could be obtained in vivo we adopted a two-step channel alignment procedure. Firstly, coarse alignment was performed using beads or an etched slide and the Softworx alignment software. This was then refined using a strain in which tet operators are bound by both tetR-GFP and tetR-mCherry. The mean deviations of the centres of the red and green foci in three planes were used to generate a vector which was then applied to all red-channel frames. This reduced the mean measurement error from 110 nm to 63 nm (Fig. 1d). This reduction is likely due to the fact that the vector generated in the colocalising strain factors in differences in refractive indices between the objective lens and the subject being viewed (media and cells). A histogram of measurements from the coarse- and fine-tune-aligned colocalising strain is presented in Additional file 1: Figure S1. As the signal to noise ratios (SNRs) of the fluorescent foci of all two colour operator strains were similar to those of the colocalising strain, and as all satisfied identical quality control criteria, we consider it reasonable to assume that the mean measurement error (63 nm) is applicable to all the measurements described below.

### Non-linear relationship between physical distance and genomic separation

**Fig. 2:**
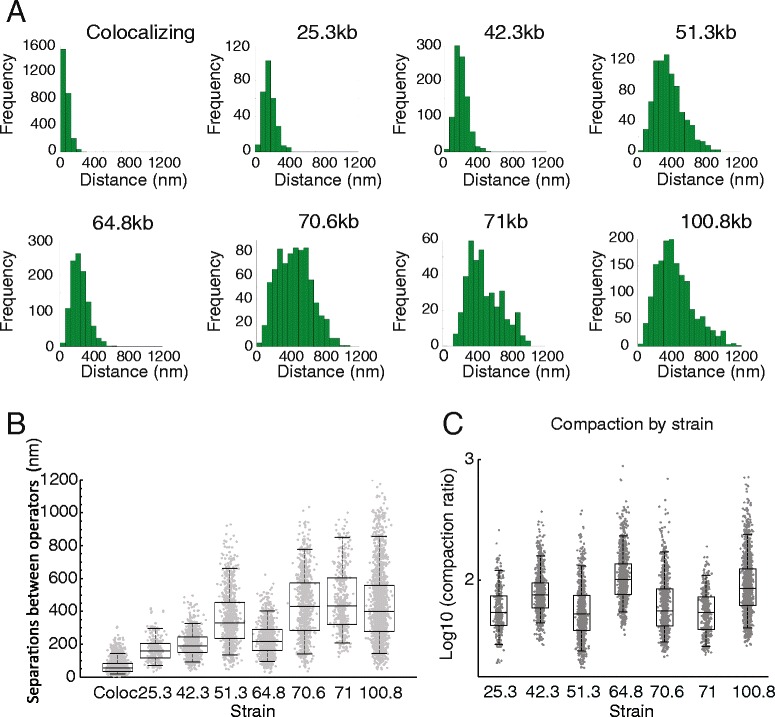
Relationship between genomic separation and physical distance. **a** Frequency distributions of spot distances for the genomic separations indicated. Bin sizes are based on mean measurement error (63 nm). **b** Box plots representing spot distances for measurements from each strain. **c** Compaction ratio defined as 0.34α/*d,* where α is the genomic separation (in base pairs) and *d* is the measured distance (in nm)

Using the workflow described above it was possible to measure the distance between two fluorescently tagged loci over time in several strains. Spot distance behaviours from all videos are presented in Additional file 1: Figure S2. Distance measurements for strains with varying genomic separations in G1 of the cell cycle are presented as histograms in Fig. 2a. When the distributions obtained from all strains are plotted in boxplot format, it is apparent that for the longer genomic separations there is a progressive but non-linear increase in the physical distance (Fig. 2b), similar to that previously reported [54].

**Fig. 3:**
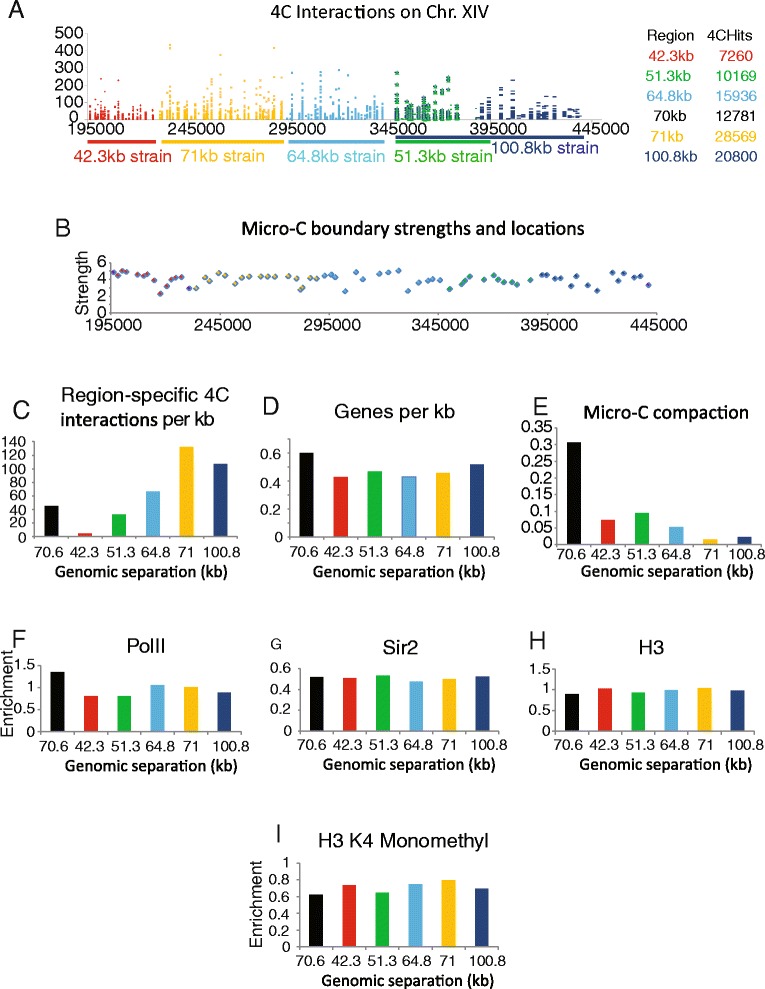
Capture contacts and chromatin composition do not correlate with compaction. **a** Distribution of Hi-C contacts [95] across a region of chromosome XIV. The different genomic separations are colour coded along with the contacts measured across each region. The total number of contacts identified within each region is indicated to the right. **b** Strengths and locations of boundaries as determined by Micro-C [56]. The same colour coding is used to identify boundaries of a given strain. The number of region-specific 4C interactions per Kb (**c**) [95], number of genes per kb (**d**), compaction measured by micro-C (multiplied by −1) (**e**) [56], mean enrichment for PolII (**f**), mean enrichment for Sir2 (**g**) [96], mean enrichment for histone H3 (**h**) [96] and mean enrichment for histone H3 monomethylated at lysine 4 (**i**) [91] are plotted for each strain with the genomic separation between operators indicated. No factors were identified that correlate well with compaction. The distribution of additional factors across this region is plotted in Additional file 1: Figure S3)

The physical separation distance can be normalised for the genomic separation and expressed as compaction (Fig. 2c). This shows that the 42, 64 and 100 kb strains are more compact than the other strains (Fig. 2c). This suggests that locus specific effects may influence chromatin compaction in addition to the genomic separation. One potential explanation for the increased compaction of the 64 kb locus is that it participates in a more extensive network of looping interactions. Chromatin capture analysis has been used extensively to monitor looping interactions. As 4C and Micro-C data have been collected for the whole yeast genome under growth conditions comparable to those we have used, it is possible to investigate the frequency of interactions observed across the chromosomal regions we studied. 4C [55] and micro-C [56] interactions are plotted across a region of chromosome XIV (Fig. 3a–c, e). The highest density of 4C interactions falls within the 71 kb strain (Fig. 3c), which is not anomalously compact (Fig. 2b). The more compact 64 kb locus is not shown to have an increased density of interacting loci. Similarly, total contacts and boundaries detected by micro-C do not correlate with the compaction observed by imaging (Fig. 3b, e).

With rich data describing the distributions of many different chromatin features being available for budding yeast, we sought to determine whether any other factors correlate with the compaction observed at the loci we have studied. Chromatin immunoprecipitation (ChIP) enrichments for 18 different chromatin features including histone modifications, histone H3 occupancy, general transcription factors and RNA polymerase, were plotted across chromosomes XIV and IV (Additional file 1: Figure S3; S5). None of these factors correlate well with the higher compaction observed in the 42, 64 and 100 kb strains. The distributions of RNA pol II, Sir2, Histone H3 ChIP, and Histone H3K4 monomethylation are shown as examples (Fig. 3d–g).

### Anisotropy is increased for large separation distances

**Fig. 4:**
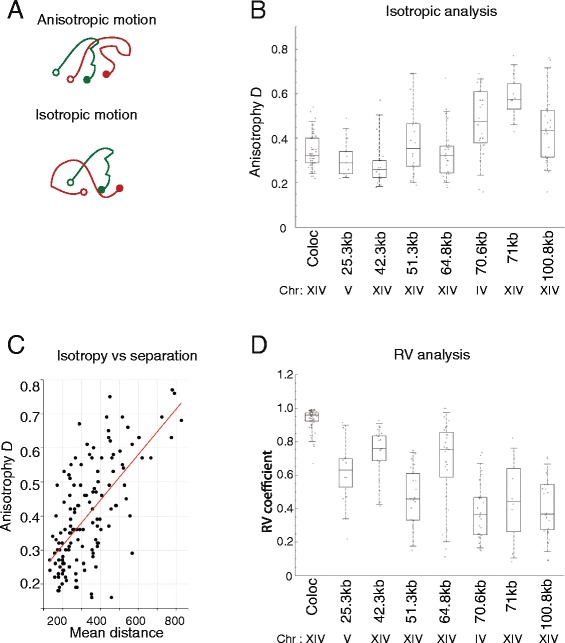
Motion analysis for strains with different genomic separations. **a** When the relative orientation of two particles is maintained as they proceed along their trajectories (indicated as lines) their motion is considered isotropic (*bottom*), when orientation changes over time motion is anisotropic (*top*). A statistic *D* was calculated as a measure of anisotropy (Materials and Methods; Fig. 4). Larger D values correspond to greater anisotropy. *D* is observed to generally increase with genomic separation (**b**). In **c** the mean *D* is plotted against the mean distance (*R* ^2^ = 0.43). **d** To assess the correlation of the direction of motion of the two operators flanking a locus, comparisons of the vectors describing the motion of each operator were made at each time point. An RV coefficient was calculated for this purpose. The general trend observed was a decrease of motion correlation as the genomic separation between the operators increased. However, for the 64 kb and 42 kb strains which adopt a more compact configuration, the trend was not observed

During imaging, it was noticeable that the relative orientations of the tagged loci in a subset of cells were markedly constrained. To analyse this quantitatively we developed a test which assigns a statistic, *D* (see Materials and Methods), which quantifies the anisotropy of the spatial orientation of the two-spot system. The higher *D*, the greater the degree of anisotropy. Lower *D* values were generally observed for genomic separations below 70 kb (Fig. 4b) and anisotropy is correlated with the mean distance (Fig. 4c). This could be an indication that for small physical distances that relative motion of foci is less constrained. As physical distance increases, the nuclear environment acts as a constraint restricting the extent of relative motion.

### Analysis of independence of motion

If two loci are rigidly coupled, then their motion is anticipated to correlate over time. In contrast, if two loci are distant and elastically connected, their motion is expected to be independent. We estimate the spatial correlation of the two dots by the RV coefficient, a multivariate extension of the Pearson’s correlation coefficient [57]. There is a weak trend for independence of motion to increase with increasing genomic distance (Fig. 4d). The exceptions to this trend are the relatively compact 64 and 42 kb genomic separations which adopt a more compact state (Fig. 2c). The increased spatial correlation at short separations could arise from a more rigid coupling across shorter intervening genomic separations, or as a result of concerted motion of chromatin within local territories within the nucleus.

### Heterogeneity in chromatin configuration between single cells persists for minutes

**Fig. 5:**
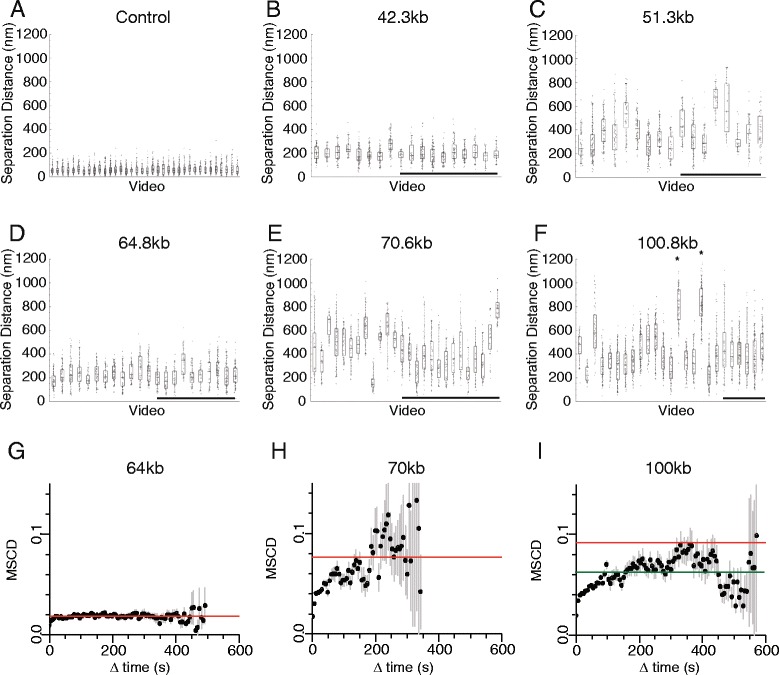
Variations in the motion of reporter loci in single cells. **a**–**f** Box plots of distances for individual cells. Non-underlined boxes indicate data collected from videos of approximately one minute in length during which Z stacks were collected approximately once per second. Underlined boxes were collected over 10 min with each Z stack collected at 6 s intervals. Variation in median distance between cells is observed, especially for the 51, 70 and 100 kb separations, in some cases over durations of up to 10 min. The the mean square change in distance (MSCD) as a function of the time interval was calculated for the 64, 70 and 100 kb strains where distance was sufficiently distinct from the 63 nm measurement error (**g**-**i**). To extend the range of time intervals that could be used, data from videos in which distance was measured at short intervals and at longer intervals were superposed and binned in 6 s time intervals. Error bars represent standard error. In each case a trend is observed for MSCD to increase reaching a plateau after 30–150 s. The red line shows the MSCD calculated by bootstrapping across random time points from random videos for a given strain (a cell-independent and time-independent estimate). The green line (in panel **i**) shows the same quantity found excluding data from two videos with unusually large distances (marked with asterisks in panel **f**). These observations suggest that over longer time intervals, the MSCD observed within cells approaches that observed between cells

As many videos, each of which consisting of up to 100 time points, were acquired during the characterisation of each strain, it was possible to compare fluctuations in the distance between operator sequences observed within and between individual cells within a given strain. In all cases cell-to-cell variation was observed, and the scale with which the means varied ranged from below 2-fold to over 4-fold for the longer genomic separations (Fig. 5a–f). From visual inspection of individual distance versus time traces it is clear that in some cases the separation distance remains relatively constant but distinct between cells (Additional file 1: Figure S2.2 fff). In other cases, a single transition is observed during the course of a movie (Additional file 1: Figure S2.2 uu), while in other cells a series of rapid fluctuations in distance is observed over short time intervals (Additional file 1: Figure S2.2 ii). Differences in mean distance were observed over both 1 min. and 10 min. (Fig. 5a–f). To assess the time scale over which distance varies, the mean square change in distance (MSCD) was plotted for different time intervals. This can be interpreted as one-dimensional mean square displacement, where changes in distance between the spots is squared rather than simply changes in position squared are plotted (Fig. 5g–l). These curves indicate that MSCD typically increases over time intervals up to 30 s. However, beyond 150 s, a plateau is reached at which little additional change in separation distance is observed. The magnitude of this plateau for MSCD varies considerably between strains with the value in the 64 kb strain being 3–4 fold less than observed in the 70 and 100 kb strains.

If the observed changes in distance occur as a result of vibrational motion that reaches a maximum by 150 s, then we would expect the distance after long time intervals within one cell to be comparable with the variation observed between cells. To test this, time independent distance changes between randomly selected time points in different cells were calculated by bootstrapping.

This time-independent and cell-independent MSCD is plotted as a red line in Fig. 5g–l. For the 64 and 70 kb strains the MSCD measured between cells is similar to the maximum MSCD observed within cells after time intervals of greater than 150 s. However, this is not the case for the 100 kb strain, as the between-cell MSCD is greater than that measured within cells (Fig. 5l). This is likely due to the unusually large spot distance in two cells in this strain (marked with asterisks in Fig. 5f). When the time-independent measurement between cells is recalculated omitting these two cells, the mean is a better fit to the value observed at time intervals of greater than 150 s within cells (green line; Fig. 5g). Large differences in mean distance were also observed in the 70 kb strain, even in videos acquired over 10 min (Fig. 5e). However, in this case these differences in separation are evenly distributed around the mean. The observation that the mean separation distance between the same loci can be distinct in specific cells for several minutes raises the possibility that there may be mechanisms acting to maintain different conformations in a subset of cells.

### Differences in chromatin conformation are not inherited through cell divisions

**Fig. 6:**
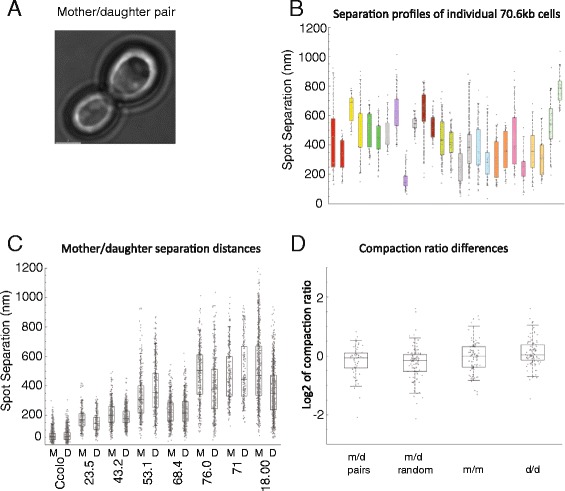
Chromatin conformation is not inherited. Mother and daughter cells can readily be identified in *Saccharomyces cerevisiae* cultures as physically attached pairs (**a**). Each mother-daughter pair shares the same parent providing an opportunity to assess whether chromatin configuration is conserved through cell division. Movies obtained in individual cells from the 70 kb strain are plotted with mother-daughter pairs in the same colour in (**b**), in each case with data from the mother to the left. Unpaired cells are in grey. In some cases it is clear that mothers and daughters have differing spot distances. The distance box plots for all mothers and daughters of each strain are plotted in (**c**). In order to compare changes in distance between a large sample of related and unrelated mother and daughter cell pairs, changes in compaction were calculated using movies from each genomic separation (**d**). Compaction is calculated by dividing genomic separation by the measured distance between operators. The change in compaction between different populations was then calculated as a ratio indicated on the y-axis as ‘Log2 of compaction ratio’. This comparison could be made between related m/d pairs and unrelated m/d pairs selected at random. In both cases, the distributions of the differences in compaction are similar and not statistically significant (Mann-Whitney *p*-value = 0.181). This indicates that the separation of the reporter loci studied here is not inherited. Little change in separation is observed when the comparison is made between unrelated mother/mother and daughter/daughter pairs. This suggests that the difference in the volume of mother and daughter cell nuclei has little effect on the separation of these operator tagged loci

Within cultures of *Saccharomyces cerevisiae*, following cell division, mother and daughter cells remain associated and can readily be identified as larger mother cells associated with a smaller daughter cell (Fig. 6a). The ease with which related siblings can be identified cytologically provides an opportunity to investigate whether chromatin state is inherited through cell divisions.

Representative histograms of paired mother and daughter cells are shown for the 70.6 kb strain in Fig. 6b. From this it is clear that chromosome conformation varies in several mother daughter pairs indicating that in these cases separation distance was not conserved through cell division. The mean spot distances observed in mother and daughter cells are similar for each strain (Fig. 5c). When changes in compaction are measured for paired mother daughter cells and compared to that in randomly selected unrelated mother daughter pairs across all strains, the means and standard distributions are similar (Fig. 6d). In addition the mean distance changes observed in comparisons between unrelated mother and daughter cells are similar (Fig. 6d), indicating that the reduced volume of the nuclei of daughter cells does not affect the variation in distance.

## Discussion

Recent developments in fluorescent microscopy provide new opportunities to study the organisation of native chromatin in live cells. Here we describe a workflow that enables live cell 3-D two-channel measurements to be made with a mean measurement accuracy of 63 nm. A key step in this workflow was the adoption of a channel alignment control that takes into consideration the optical properties of the experimental sample. Although our application of this approach was based in yeast cells, similar two colour alignment using two versions of a fluorescent reporter is applicable to many cell based systems. It is likely that improvements in several aspects of the work flow will further reduce measurement error. As the greatest error component is along the axial dimension, approaches such as confocal microscopy, multiphoton microscopy, or optical astigmatism would all likely decrease mean measurement error.

We use this system to study the configuration of chromatin across a series of chromosomal loci with distinct genomic separations. In all cases the physical distance between the fluorescent reporters is observed to fluctuate over time. In addition, a trend is observed for distance to increase at larger genomic intervals. The scale of the measurements is comparable to data obtained previously using FISH, 2D [54] and more recently 3D data [34]. Information of this type can be used to test different physical models describing chromatin fibres.

At longer distances above roughly 300 nm (corresponding to a genomic separation of approximately 70 kb), we observe that the two reporter loci have a higher tendency to remain in the same relative orientation with respect to one another. Structure within the nucleus may restrict diffusive motion on this scale. At the same time there is greater correlation of motion of loci at closer proximity than ~300 nm. This could arise as a result a physical coupling between the two operators. It is also possible that the local chromatin environment extending to 300 nm, a form of sub chromosomal territory or domain, undergoes some localised flow-like motion that accounts for the correlated motion. On scales greater than 300 nm the density of intervening chromatin may act to exclude free diffusion and restrict isotropy. At these larger distances, the motion of the two operators becomes independent as indicated by the reduced RV coefficient. The differences in motion observed over different ranges may relate to previously reported effects of nuclear exclusion on chromosome organisation [58].

The measured separation distance did not vary consistently with genomic separation across all the strains studied. The 42, 64 and 100 kb strains show higher levels of compaction than the other strains (Fig. 2c). When the diffusive motion of the lac and tet operators flanking these loci is studied independently, the mean square displacement curves are similar (Additional file 1: Figure S4). This suggests that the differences in compaction do not result from constraints to the motion of either of the reporter sequences. Another potential explanation for the reduced separation distances observed in the 42, 64 and 100 kb strains could be that chromatin is arranged in a more compact state over these genomic loci. To investigate this we took advantage of the large number of previously published genomic datasets available in budding yeast and searched for factors that correlated with chromatin compaction across the different loci we have studied. Amongst the 18 factors selected (including histone occupancy, post-translational modifications and measures of transcriptional activity) none show a strong correlation with compaction (Fig. 3; Additional file 1: Figure S3). In addition, the loci within these strains are not adjacent to loci such as centromeres, telomeres or the rDNA locus that have previously been observed to influence subnuclear motion [38].

Looping interactions that affect the separation of reporter loci provide an attractive explanation for the variations in the observed separation distance both between strains and over time. Hi-C approaches are widely used to detect looping interactions. Like microscopy-based approaches they detect heterogeneity in chromatin conformation between cells [59] and can be used to model chromosome architecture [55]. However, the distributions of cross-links obtained by Hi-C, and high resolution micro-C, do not correlate with the higher compaction observed in the 42, 64 and 100 kb strains (Fig. 3). This is perhaps not surprising as although these loci are relatively compact, the distance between operators is typically 200 nm which is beyond the range likely to enhance DNA ligation, the readout for capture based approaches. Chromosomal loci can potentially be constrained through interactions with any relatively immobile object within the nucleus. Such interactions may bring heterologous DNA sequences into closer proximity but still out with the range required to enhance ligation. Factors such as these are likely to contribute to the previously noted discord between Hi-C and imaging based chromatin measurements [60]. Where chromatin is especially well ordered there is a greater chance that imaging and Hi-C approaches converge. An example of this is provided by the mating type loci on budding yeast chromosome III [61].

Changes in the association of loci with relatively immobile bodies within the nucleus that affect the distance between reporter loci provide an attractive means of accounting for some of the heterogeneity we have observed. Such interactions could be stable over differing time scales. Transient interactions could account for the rapid variation in distance observed at some loci (Additional file 1: Figure S2). Where interactions are more stable they could contribute to variation in the mean distance observed in different cells. A diverse range of factors could act to influence the localisation of a given chromosomal locus and this could explain why no obvious correlation between genomic features and separation distance was identified.

The use of *Saccharomyces cerevisiae* makes it relatively easy to identify pairs of cells that share a common mother. Comparing the conformation of chromatin between related and unrelated mother daughter pairs, chromatin conformation was not observed to be conserved in related cells. However, it is possible that this will not be the case for all loci. In budding yeast it is well established that expression of genes at the mating type loci [62] and within subtelomeric regions [63] can be inherited. Furthermore the nuclear localisation of these regions is distinct [61, 64]. The chromosomal regions we studied do not include these regions which may be exceptions within the context of *Saccharomyces cerevisiae* genes. Higher eukaryotes possess additional chromatin features such as HP1 proteins and polycomb that are more likely to influence both inheritance and nuclear localisation [65, 66]. Consistent with this, inducible decompaction of reporter loci in mouse embryonic stem cells has been observed to be sufficient to cause a change in subnuclear localisation that persists through cell divisions [67].

Although we do not observe any evidence for the inheritance of chromatin configuration through cell division, we do observe individual cells that have distinct chromatin configurations that persist for times periods of up to 10 min. This suggests that alternate chromatin configurations can be maintained in individual cells. It’s possible that this heterogeneity may affect the ability of cells to respond to environmental stimuli. There is good evidence indicating that the subnuclear localisation of genes can play an important role in their regulation. Tethering genes to the nuclear periphery is known to favour establishment of silent heterochromatin [68, 69], many genes have been observed to transiently associate with the nuclear pore during activation [70]. In mammalian cells changes in the localisation of genes has been observed to correlate with changes in transcription [71–73]. If the conformation of loci has a similar influence on gene regulation, then it could contribute to the heterogeneity in transcriptional responses that have been observed in single yeast cells [74–76]. This heterogeneity potentially provides an advantage for individual cells in being able to respond rapidly to an environmental change. However, unlike the changes occurring during the development of multicellular organisms there is not a need for such changes to be inherited. Instead, if a cell is well placed to adapt to a new environment it may be best to restore heterogeneity in subsequent generations providing capability to respond rapidly to a diverse range of future challenges. As we do not observe evidence for the inheritance of chromatin configuration at this level, it is possible that the processes of DNA replication and cell division provide an opportunity to reset the configuration to the spectrum of states observed within the population. In this way non inherited heterogeneity may serve an important biological function. In multicellular organisms there is a need both for the flexibility to respond to environmental change and the precision required for tissue development. This may involve a different balance between inherited and non-inherited states.

## Conclusions

A workflow has been established to study the separation of fluorescently tagged reporter loci in live cells. The mean separation of reporter loci was observed to increase with increasing intervening genomic sequence. However, this increase is non-linear indicating that different regions of the genome are in different configurations. No genomic features were identified that correlate with the observed separation of loci suggesting that as yet uncharacterised factors influence chromosome organisation. Separation is observed to vary within cells over time and between cells. This heterogeneity may contribute to heterogeneity in the transcriptional response at the level of single cells. Distinct chromatin configurations were however, not observed to be inherited through cell division.

## Methods

### Plasmids and strains

The plasmids used in this study are summarized in Additional file 2: Table S1. Plasmids pT1196 [77], pFA6a-mCherry-natMX6 [78], pAT253corrected [52], pLAU43 and pLAU44 [79], pRS416 [80], pAFS59 [32], pAFS135 [81], pRS306tetO224 [82], pYiplac204-Gal1Pro-MDN1 [83], pGVH30 [54], pFA6KanMX6 [84] and pAG25 and pAG32 [85] have been described previously. pDD2244 (tetR-GFP-tetR-mCherry::ADE2) was generated in 4 cloning steps from pAT253corrected (Taddei), pT1196, and pKS391. pDD2245 (GFP-lacI**-TetR-mCherry::ADE2) was generated in 4 cloning steps from pKS391, pGVH30, pAG32, and pAT253corrected. pDD2246 (tetO-UBP10::TRP1) was generated from pLAU44, an NdeI-UPB10 integration site-HindIII PCR product, and an AatII-TRP1-NdeI PCR product fragment in 2 cloning steps. pDD202 was generated in 5 cloning steps from pRS306tetO224, pRS416 and 3 PCR products. pDD206 was generated in 4 cloning steps from pYCG_YLR106c, pRS416, and pFA6KanMX6. pDD207 was generated in 4 cloning steps from pYiplac204-Gal1Pro-MDN1, pRS416, and pAFS59. The lac operator array plasmids pDD249, pDD251, pDD253, and pDD254 used to generate yeast strains DD1471-1475 were constructed by cloning the appropriate genomic integration target sequence from Chr XIV adjacent to the lac operator array in a pLAU43 lacOx240 clone which had previously been modified with a SalI-URA3-SalI PCR product fragment. The tet operator array plasmids pDD2246, pDD250, pDD252, pDD255, pDD256 and p2577 used to generate strains DD1471-1475 were constructed by cloning a AatII-TRP1-NdeI PCR product fragment into the pLAU44 tetOx240 plasmid and then cloning the appropriate genomic integration target sequence from Chr XIV adjacent to the tet operator array. All plasmid sequences are available upon request. All plasmids were verified by multiple restriction digests as well as sequencing of crucial regions.

The *S. cerevisiae* strains in this study are summarized in Additional file 2: Table S2 and illustrated in Fig. 1a. The tet and lac operator arrays for all Chr XIV strains were integrated between convergent genes. The terminators of these genes were duplicated and flank the insertion sites such that all genes retain their wild type terminators. Additional file 2: Table S2 columns 5’ and 3’ indicate the pairs of convergent genes where the insertions took place. DD1407 generation: WT yeast strain K699 (W303) was transformed first with pDD2244 (tetR-GFP-TetR-mCherry::ADE2) linearized with PciI, and then with pDD2246 linearized with PfoI. DD1413 was generated by successively cloning in linearized pAFS135, pDD2248, pDD202, pDD206, and pDD207. It was verified by PCR using primer pairs 1988 + 1952, 2061 + 2062, 2044 + 2529, and 2051 + 2058, which flank the appropriate integration sites at the URA3 locus. Strains DD1471-1475 were generated using plasmids pDD2244 and pDD2245, and the appropriate lacO and tetO array plasmids pDD246, 2247, 2248, and pDD249-256, and were verified by PCR using primers 2452–2468. Strain DD1336 was generated by cloning linearized pDD2247 into T6002 and was verified by PCR using primers 2067–2074.

### Cell culture

Tetracycline was added to all cell cultures to diminish the affinity of the tet repressor DNA binding protein for its DNA binding site, as per Dubarry [52]. Optimal concentration resulting in 94 % maximum fluorescence intensity was determined via concentration series and measured on the OMX. The colocalising strain and most sample strains were streaked to YPAD and cultured overnight, propagated in liquid culture for 8 h, cultured overnight in 75%SC/25%YPA + 2%dextrose + 20 ng/ml tetracycline, washed in the culture media and placed on ice. In all cases cells were adhered to concanavalin A-treated 35 mm glass-bottomed MatTek culture dishes for 10 min at 22.0 °C, then allowed to temperature-equilibrate in the microscope enclosure at 23.5 °C for an additional 10 min.

### Image acquisition

All imaging was performed with a GE|OMX Blaze® microscope. Immersion oil with refractive index 1.514 was used in all cases. Typical video acquisition included 5 μm stack height, 250 nm step size, 21 images per stack, 128 × 128 field of view (FOV), 50–100 time points, excitation 3–8 msec, ND 31–100 for mCherry and ND 5–10 for GFP, and sequential channel acquisition. The microscope enclosure is maintained at 23.5° Celsius. Videos and associated tracking data are available at url: http://dx.doi.org/10.17867/10000102.

### Channel alignment protocol and image processing

Channel alignment parameters for an initial coarse alignment were generated using single stacks of twenty-nine 1024x1024 FOV images with step size of 125 nm of a Tetraspeck 100 nm bead slide or laser milled slide (LMS) using the red and green channel cameras. Coarse alignment lateral parameters were calculated using Softworx software, which included a translation, magnification, and rotation. Coarse axial alignment offset was determined manually using the Softworx Measure Chromatic Correction function. Channel alignment fine tuning parameters were calculated using live cell 3-D tracking data from the colocalising strain, DD1407, which has single red and green colocalising spots of dimensions assumed to be smaller than the PSF. 50–100 time points of 17 128 × 128 FOV images/stack were generated with 3-8 msec exposures generated sequentially from red and green channels. Videos were split into individual channels and saved in 4-byte float format. Darkfield images, generated previously by taking the mean pixel intensity of 1000 images at set exposure times, were then subtracted from the individual video channels to correct for noise arising from the CMOS cameras. Videos were denoised with the ND-SAFIR denoising software [86] using the Sedat Lab settings iter = 5, p = 3, sampling = 2, noise = Gaussian, adapt = 0, island = 4, and np = 8 [53], and then deconvolved via Softworx using a ratio (conservative) method tailored to an idealized objective of the model used in our OMX Blaze, and saved in 4-byte float format. Individual channel files were fused into a single red plus green video file and coarse alignment was performed with Softworx using the bead slide or LMS alignment offset parameters. The spots were tracked using Imaris, the x, y, and z offsets between the different channels were determined for each time point using Excel spreadsheet, and the means of these offsets were calculated from data from multiple videos. Fine-tuning alignment was performed in Excel by subtracting these mean offsets from the red channel x, y, and z spot coordinates, which resulted in a final translation. After the fine tuning alignment had been performed on the colocalising strain videos, the mean distance between the red and green spots for all time points from all videos was determined via Pythagorean Theorem to be 63 nm, with a standard deviation of 37 nm. The tracking data is available at https://idr-demo.openmicroscopy.org/webclient/annotation/1645869.

### Quality control

Plotting z coordinate versus error indicated that once tagged loci diffused to within 1 μm of the top or bottom of a stack, error increased (not shown), and for this reason these data points were eliminated from both control and sample data sets. Plotting maximum spot intensity or contrast versus error, followed by LOESS smoothing, revealed correlations which allowed for elimination of data points with high error (not shown). Based on this GFP and mCherry contrast thresholds were both set at 12, while minimum intensity threshold for mCherry and GFP was set at 25 and 16 respectively. Use of these thresholding values removed images where the positions of foci were not sufficiently well defined to obtain high resolution locations. Using these threshold values, we were able to generate live cell 3-D 2-channel videos with 250 nm step size, 5 μm stack size, 21 images per stack, and up to 100 time points. As experimental video data were subjected to the same thresholding protocol, it is assumed that the resulting error was also 63 nm.

### Relative orientation anisotropy

We assessed the degree of anisotropy of the spatial orientation of each pair of marked loci by mapping them to UV-space. The Cartesian co-ordinates ***r***_*i*_ = (*x*_*i*_, *y*_*i*_, *z*_*i*_) of the distance vector between the ends of a locus were mapped into a unit square,

1 $$ \begin{array}{l}{u}_i=\frac{1}{2\pi }{ \tan}^{-1}\frac{y_i}{x_i},\hfill \\ {}{v}_i=\frac{\left|{\boldsymbol{r}}_i\right|-{z}_i}{2\left|{\boldsymbol{r}}_i\right|}.\hfill \end{array} $$

If the original vectors ***r***_*i*_ are isotropic (uniformly distributed on a sphere ${\mathbb{S}}^2$), then the transformed co-ordinates (*u*_*i*_, *v*_*i*_) are uniformly distributed over [0, 1]^2^. We applied a two-dimensional Kolmogorov-Smirnov test [87, 88] to all (*u*_*i*_, *v*_*i*_) across each video. The test statistic, *D*, measures the degree of anisotropy in the ***r***_*i*_ distribution.

### RV analysis

RV coefficient analysis is similar to Pearson’s correlation coefficient analysis, but is a multivariate generalization rather than bivariate [57]. When used to compare the positions of two genomic loci in 3D over time, the RV coefficient indicates relative independence of motion of the two loci. Larger magnitudes of the coefficient correspond to a greater tendency for the two loci to track together.

### Mean square displacement analysis

Mean square displacement analysis was performed for individual fluorescent loci using accepted methods [89].

### The OMX Blaze microscope

The GE|OMX Blaze® microscope is fitted with an Olympus UPlanSApo 60× 1.42NA oil objective, a Piezo stage, a BGR standard filter set (DAPI 436/31, FITC 528/48, A568 609/37, cy5 683/40), conventional widefield solid-state 461–189 nm and 563–588 nm LED solid state light sources, 3 back-illuminated 15 bit scientific CMOS cameras (PCO AG, Germany) with 1024 × 1024 chip size, and a temperature control chamber set to 23.5 C. The instrument is controlled by proprietary GE software.

## Additional files

Additional file 1:
**Figure S1.** Two step channel alignment improves resolution. Channel alignment has traditionally been performed using multispectral beads of dimensions smaller than the PSF. Stacks of 100 nm Tetraspeck bead images in separate channels were analysed using Softworx alignment software to calculate rotational, translational, tilt, and magnification offsets. When colocalising strain videos were aligned following this protocol the mean measured distance between the tagged loci was calculated to be 110 nm. Including the fine-tuning step reduced the mean error to 63 nm. Error bars are standard deviation. **Figure S2.** Distance verses time plots. For individual videos acquired in this study, spot separation distance in um is plotted against time in seconds. Graphs are grouped by strain as indicated. Each video is assigned an alphabetical identifier which corresponds. **Figure S3.** Chromatin composition at reporter loci. High-resolution ChIP profiling enrichments for the chromatin constituents indicated across the loci studied. Rpb1, PolII Ser2P, PolII Ser5P, Pcf11, Spt4, Spt5, Spt6, Spt16, and TFIIB data were generated by [90]. Histone H3 K36 monomethylation, K36 dimethylation, and K79 trimethylation data were generated by [91]. Histone H2B K123 ubiquitylation data were generated by [92]. Ino80 data were generated by [93]. **Figure S4.** MSD curves for operators flanking each reporter locus. Mean square displacement is plotted for the fluorescently tagged operator sequences flanking each locus used. Data for the GFP tagged lacI are shown in green and mCherry tetR in red. Data from movies taken over different time scales is combined. The grey line indicates the profile anticipated for anomalous diffusion, *MSD*(*Δt*) ∝ *Δt* ^1/2^ [89]. The green and red lines show the best-fitting lines of Brownian diffusion, *MSD*(*Δt*) ∝ *Δt*. **Figure S5.** PolII enrichment at tetO and lacO array integration sites on Chr XIV. Enrichment of PolII subunit Rpb3 along the relevant loci is shown in green [94]. Integration sites of arrays of bacterial repressor binding sites are indicated by down carats (‘V’). Locations of open reading frames, as well as their position on Watson or Crick strands, are indicated by blue and purple arrows. (A) All lacO and tetO arrays on Chr XIV were integrated between convergent genes and the terminators of the convergent genes were duplicated such that each copy flanked the insertion site. In this way all genes retained wild type copies of their terminators after insertion. (B) PolII enrichments at the integration sites of the 70.6 kb strain. TetO array was integrated between YDL089w and YDL088c, and lacO array was integrated between YDL055c and YDL054c. (C) PolII enrichments at the integration sites of the 25.3 kb strain. This strain is flanked by the ura3-1 point mutant and wild type URA3. (PDF 5372 kb) Additional file 2: Plasmids and strains used in this study. (PDF 460 kb)

## Abbreviations

ChIP: Chromatin immunoprecipitation
FOV: Field of view
K-S test: Kolmogorov-Smirnov test
LMS: Laser milled slide
MSDC: Mean square distance change
OMX: Optical microscope experimental
PSF: Point spread function
RI: Refractive index
TAD: Topologically associated domain
