# Supplementary material for: High resolution imaging reveals heterogeneity in chromatin states between cells that is not inherited through cell division
Source: BMC Cell Biol. 2016 Sep 8;17(1):33. doi: 10.1186/s12860-016-0111-y (PMC5016949; doi:10.1186/s12860-016-0111-y)
Supplement: Additional file 1: — Figure S1. Two step channel alignment improves resolution. Channel alignment has traditionally been performed using multispectral beads of dimensions smaller than the PSF. Stacks of 100 nm Tetraspeck bead images in separate channels were analysed using Softworx alignment software to calculate rotational, translational, tilt, and magnification offsets. When colocalising strain videos were aligned following this protocol the mean measured distance between the tagged loci was calculated to be 110 nm. Including the fine-tuning step reduced the mean error to 63 nm. Error bars are standard deviation. Figure S2. Distance verses time plots. For individual videos acquired in this study, spot separation distance in um is plotted against time in seconds. Graphs are grouped by strain as indicated. Each video is assigned an alphabetical identifier which corresponds. Figure S3. Chromatin composition at reporter loci. High-resolution ChIP profiling enrichments for the chromatin constituents indicated across the loci studied. Rpb1, PolII Ser2P, PolII Ser5P, Pcf11, Spt4, Spt5, Spt6, Spt16, and TFIIB data were generated by [90]. Histone H3 K36 monomethylation, K36 dimethylation, and K79 trimethylation data were generated by [91]. Histone H2B K123 ubiquitylation data were generated by [92]. Ino80 data were generated by [93]. Figure S4. MSD curves for operators flanking each reporter locus. Mean square displacement is plotted for the fluorescently tagged operator sequences flanking each locus used. Data for the GFP tagged lacI are shown in green and mCherry tetR in red. Data from movies taken over different time scales is combined. The grey line indicates the profile anticipated for anomalous diffusion, MSD(Δt) ∝ Δt 1/2 [89]. The green and red lines show the best-fitting lines of Brownian diffusion, MSD(Δt) ∝ Δt. Figure S5. PolII enrichment at tetO and lacO array integration sites on Chr XIV. Enrichment of PolII subunit Rpb3 along the relevant loci is shown in green [94]. Integration s [file 12860_2016_111_MOESM1_ESM.pdf]

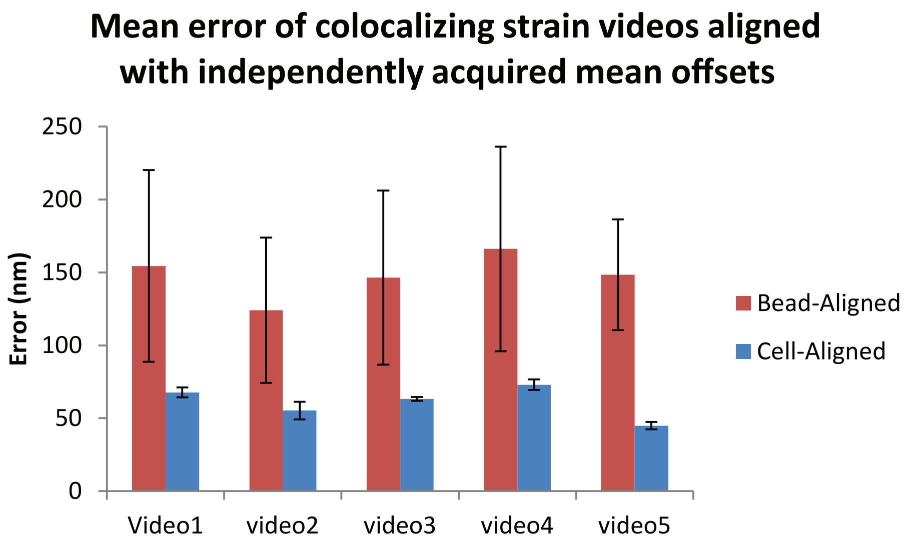
Mean error of colocalizing strain videos aligned with independently acquired mean offsets
Error (nm)
250
200
150
100
50
0
Video1
video2
video3
video4
video5
Bead-Aligned
Cell-Aligned

25.3kb

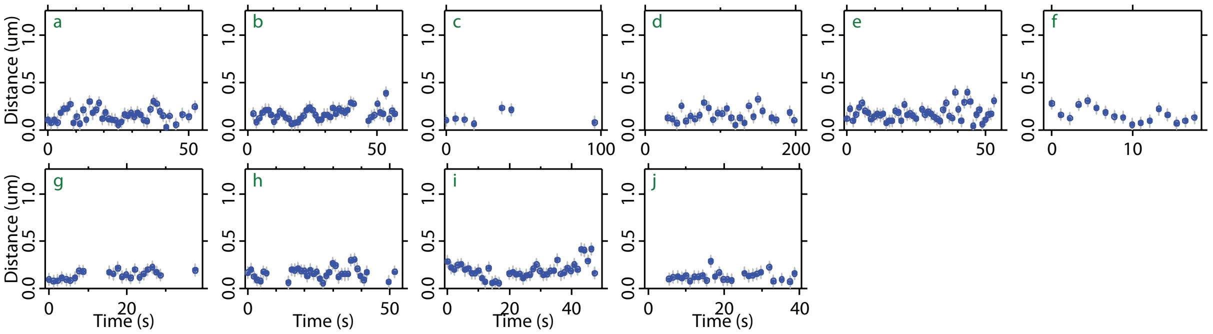

42.3kb

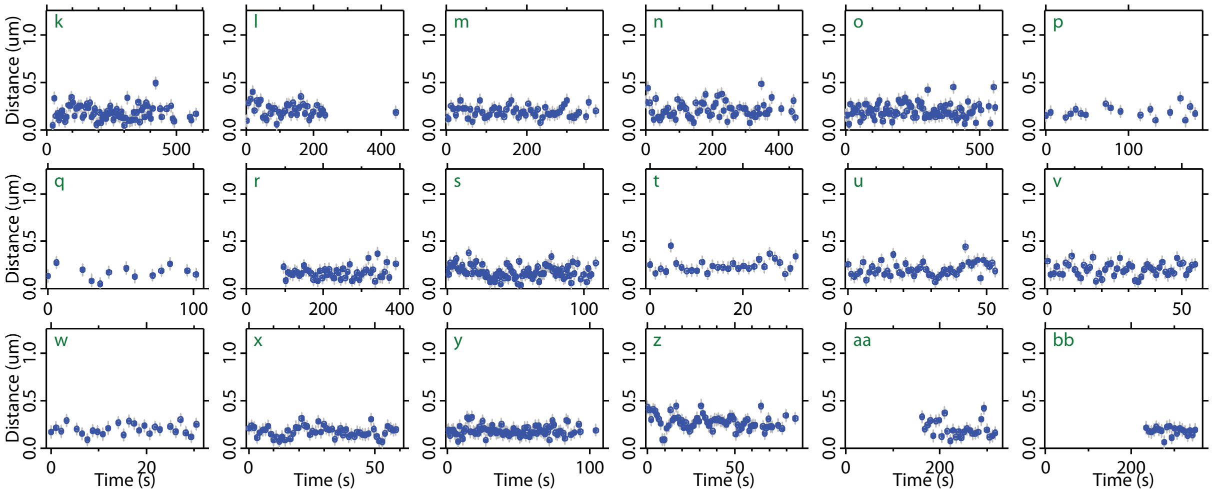

51.3kb

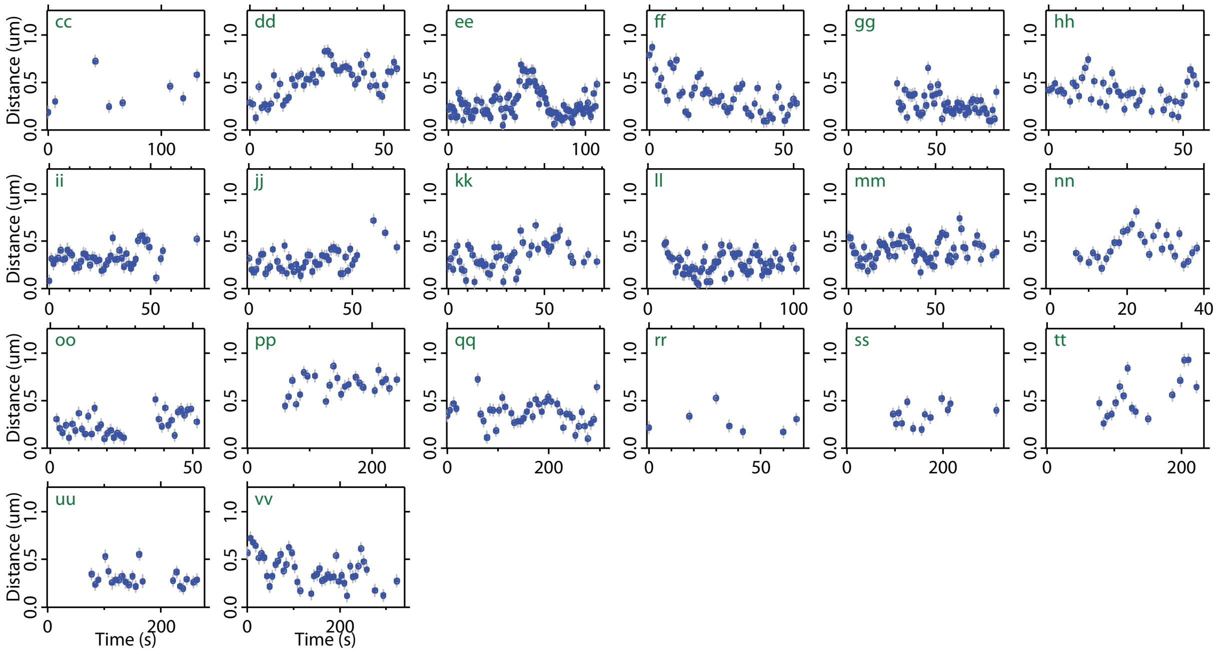

64.8kb

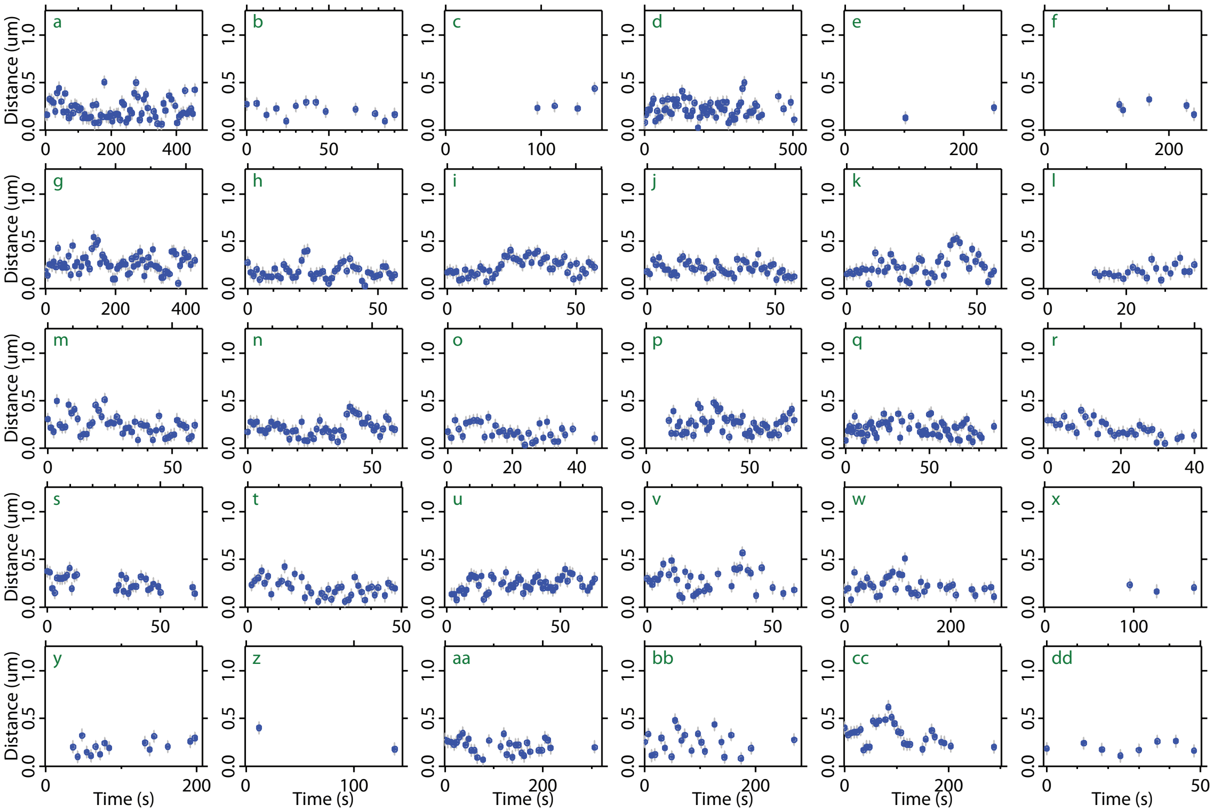
a
b
c
d
e
f
g
h
i
j
k
l
m
n
o
p
q
r
s
t
u
v
w
x
y
z
aa
bb
cc
dd
Distance (um)
Time (s)

70.6kb

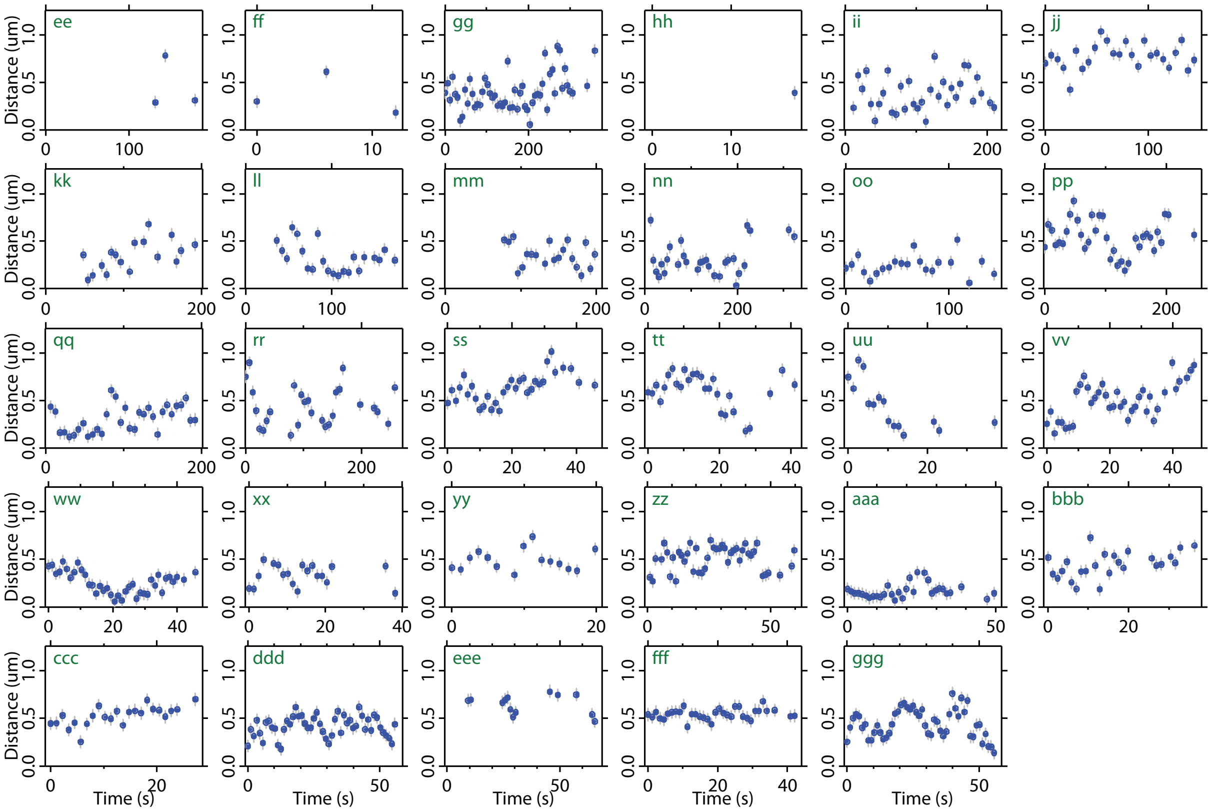
ee
ff
gg
hh
ii
jj
kk
ll
mm
nn
oo
pp
qq
rr
ss
tt
uu
vv
ww
xx
yy
zz
aaa
bbb
ccc
ddd
eee
fff
ggg
Distance (um)
Time (s)

71kb

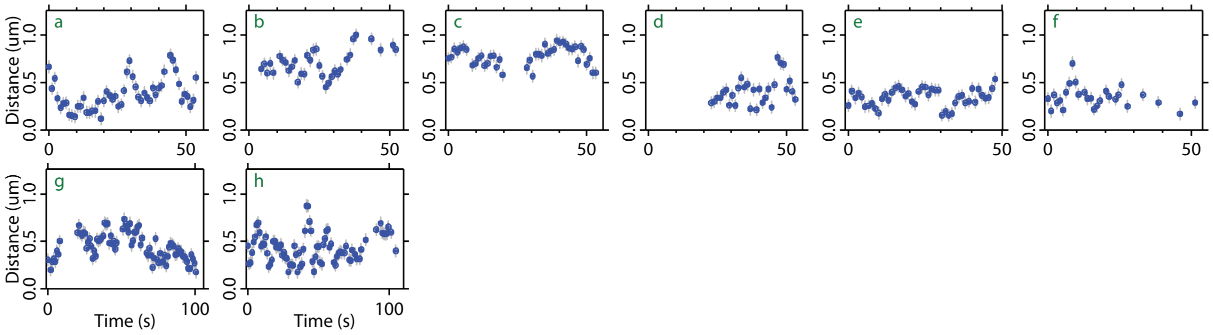
a
b
c
d
e
f
g
h
Distance (um)
0.0
0.5
1.0
0
50
100
Time (s)

100.8kb

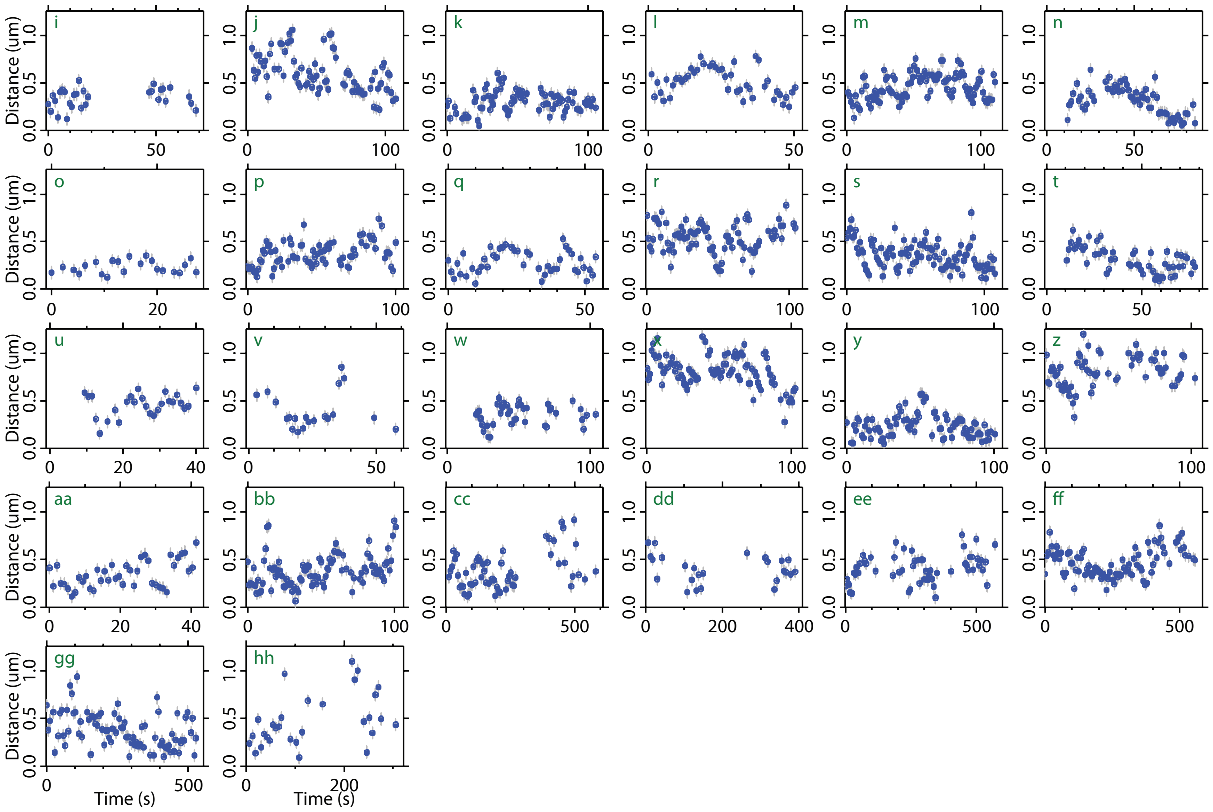
i
j
k
l
m
n
o
p
q
r
s
t
u
v
w
x
y
z
aa
bb
cc
dd
ee
ff
gg
hh
Distance (um)
0.0
0.5
1.0
0
20
40
50
100
200
400
500
Time (s)

Coloc

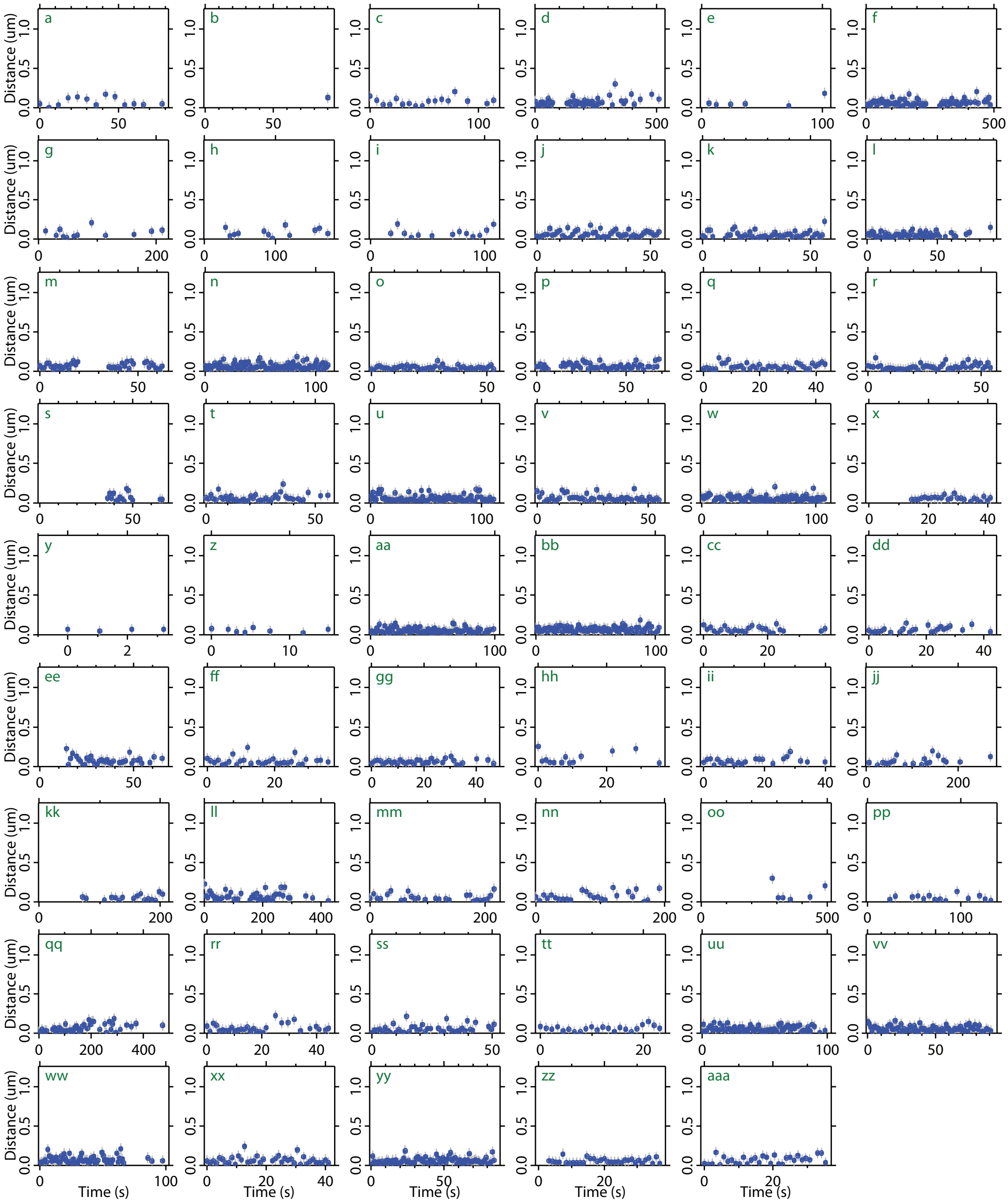
Distance (um)
Time (s)
a
b
c
d
e
f
g
h
i
j
k
l
m
n
o
p
q
r
s
t
u
v
w
x
y
z
aa
bb
cc
dd
ee
ff
gg
hh
ii
jj
kk
ll
mm
nn
oo
pp
qq
rr
ss
tt
uu
vv
ww
xx
yy
zz
aaa

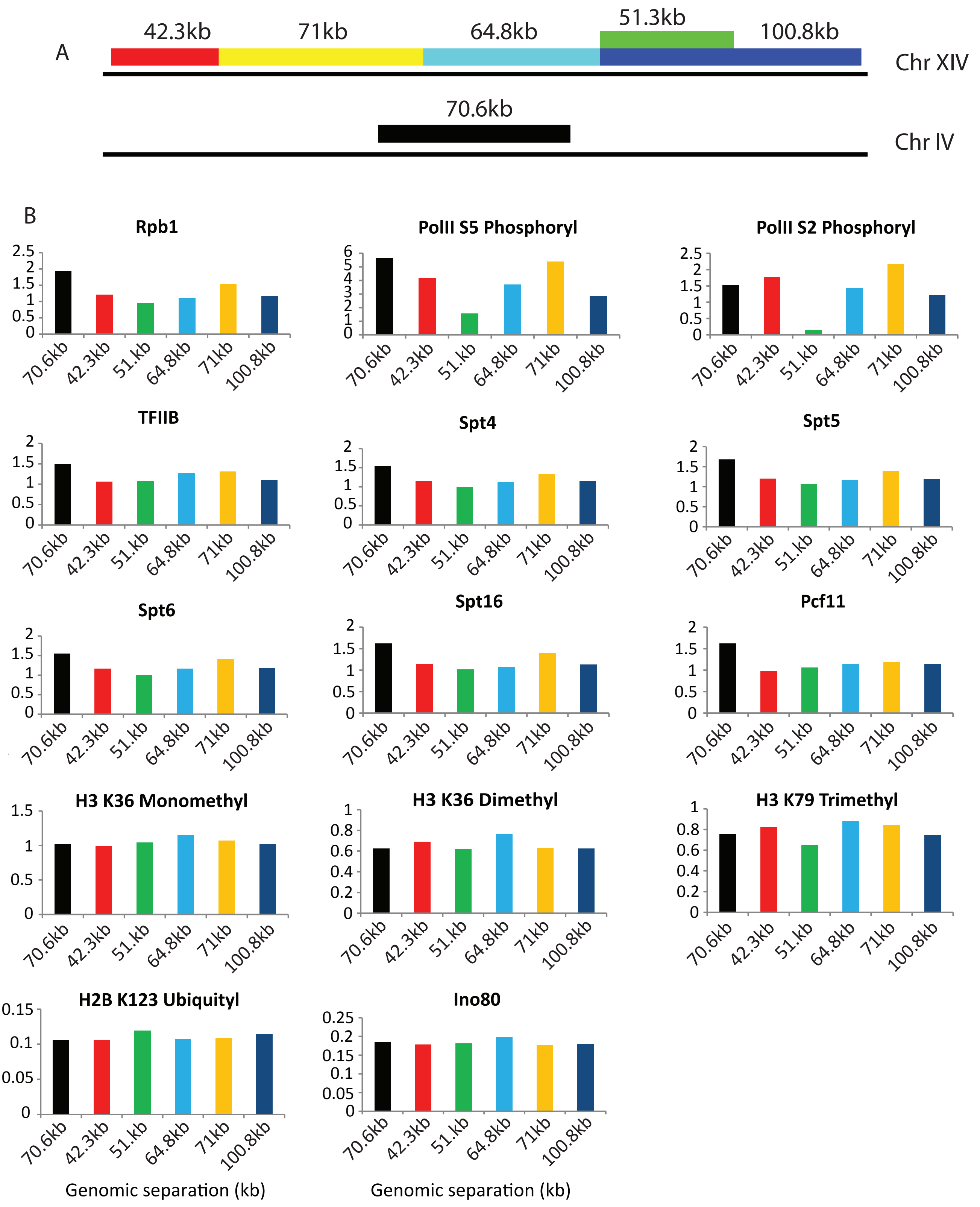
A
42.3kb
71kb
64.8kb
51.3kb
100.8kb
Chr XIV
70.6kb
Chr IV
B
Rpb1
PolII S5 Phosphoryl
PolII S2 Phosphoryl
TFIIB
Spt4
Spt5
Spt6
Spt16
Pcf11
H3 K36 Monomethyl
H3 K36 Dimethyl
H3 K79 Trimethyl
H2B K123 Ubiquityl
Ino80
70.6kb
42.3kb
51.kb
64.8kb
71kb
100.8kb
Genomic separation (kb)
Genomic separation (kb)

Dickerson et al., Figure S4

3D MSD, spots analyzed independently

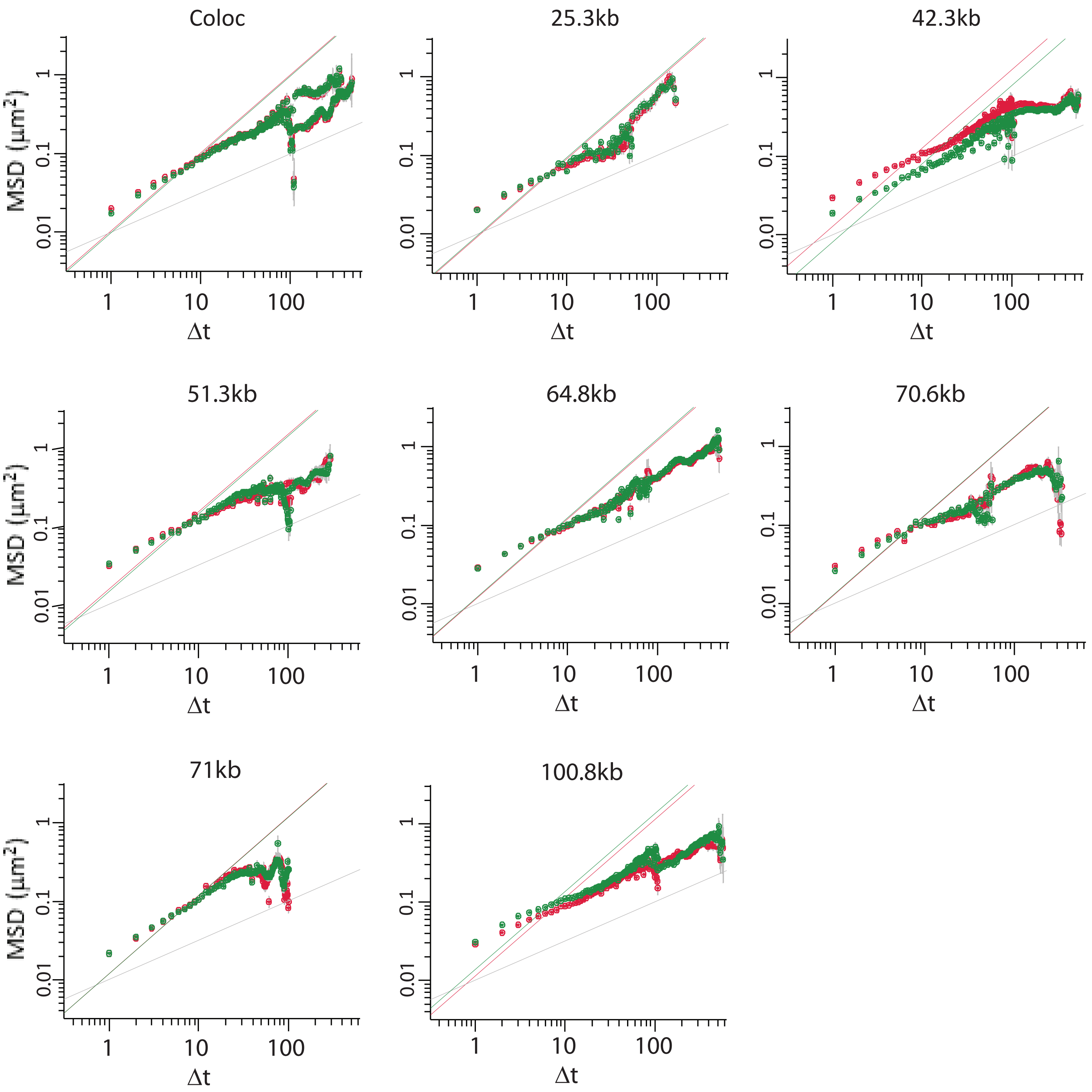

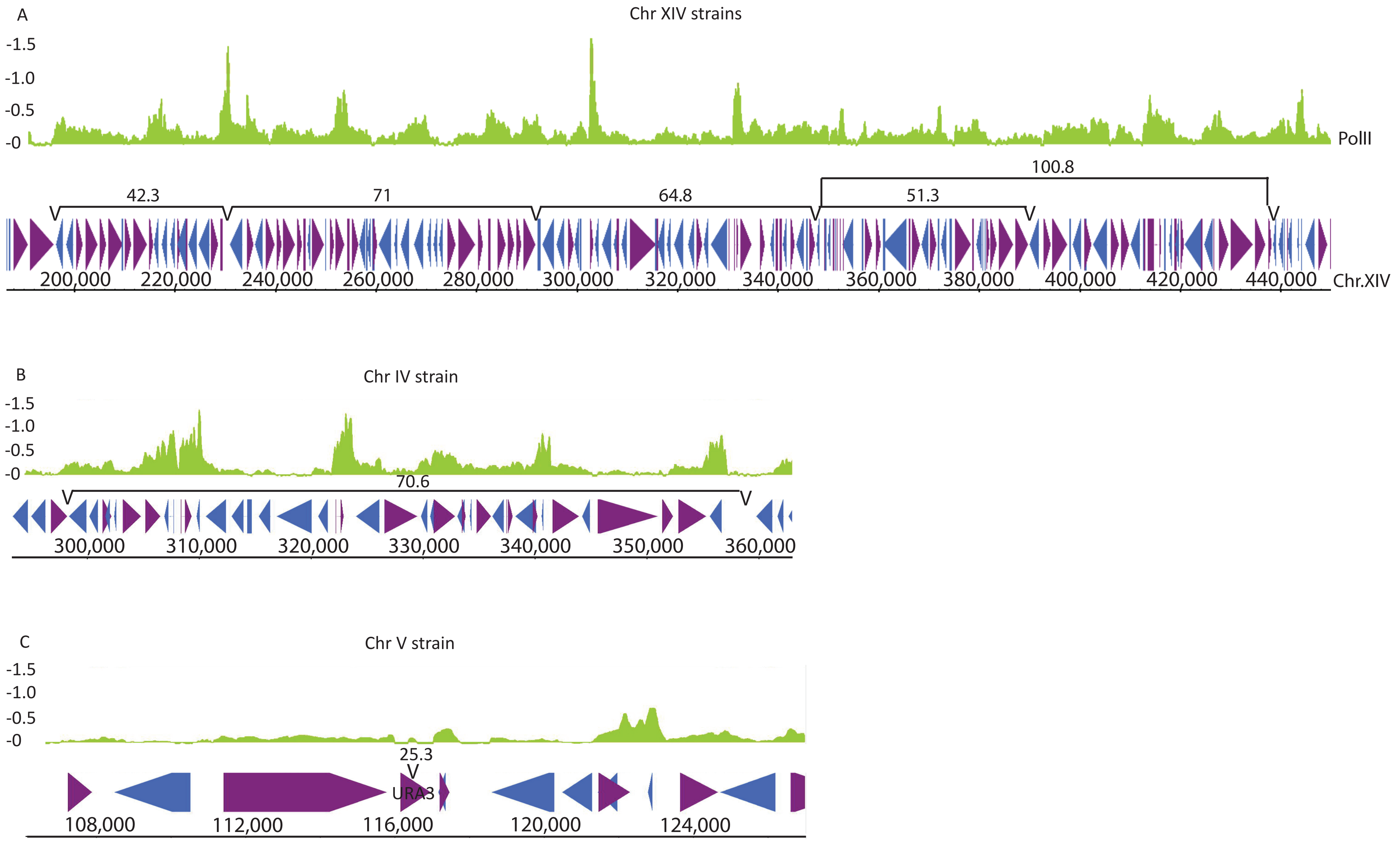
A
Chr XIV strains
-1.5
-1.0
-0.5
-0
PolII
100.8
42.3
71
64.8
51.3
200,000
220,000
240,000
260,000
280,000
300,000
320,000
340,000
360,000
380,000
400,000
420,000
440,000
Chr.XIV
B
Chr IV strain
-1.5
-1.0
-0.5
-0
70.6
300,000
310,000
320,000
330,000
340,000
350,000
360,000
C
Chr V strain
-1.5
-1.0
-0.5
-0
25.3
URA3
108,000
112,000
116,000
120,000
124,000

Fig. S1. Two step channel alignment improves resolution.

Channel alignment has traditionally been performed using multispectral beads of dimensions smaller than the PSF. Stacks of 100 nm Tetraspeck bead images in separate channels were analysed using Softworx alignment software to calculate rotational, translational, tilt, and magnification offsets. When colocalising strain videos were aligned following this protocol the mean measured distance between the tagged loci was calculated to be 110 nm. Including the fine-tuning step reduced the mean error to 63 nm. Error bars are standard deviation.

Figure S2 Distance verses time plots

For individual videos acquired in this study, spot separation distance in um is plotted against time in seconds. Graphs are grouped by strain as indicated.

Figure S3 Chromatin composition at reporter loci.

High-resolution ChIP profiling enrichments for the chromatin constituents indicated across the loci studied. Rpb1, PolII Ser2P, PolII Ser5P, Pcf11, Spt4, Spt5, Spt6, Spt16, and TFIIB data were generated by [1]. Histone H3 K36 monomethylation, K36 dimethylation, and K79 trimethylation data were generated by [2]. Histone H2B K123 ubiquitylation data were generated by [3]. Ino80 data were generated by [4].

Figure S4 MSD curves for operators flanking each reporter locus.

Mean square displacement is plotted for the fluorescently tagged operator sequences flanking each

locus used. Data for the GFP tagged lacI are shown in green and mCherry tetR in red. Data from movies taken over different time scales is combined. The grey line indicates the profile anticipated for anomalous diffusion, $MSD(\Delta t)\ \alpha\ \Delta t^{1/2}$ [5]. The green and red lines show the best-fitting lines of Brownian diffusion, $MSD(\Delta t)\ \alpha\ \Delta t$.

Figure S5 PolII enrichment at tetO and lacO array integration sites on Chr XIV

Enrichment of PolII subunit Rpb3 along the relevant loci is shown in green [6]. Integration sites of arrays of bacterial repressor binding sites are indicated by down carats ('V'). Locations of open reading frames, as well as their position on Watson or Crick strands, are indicated by blue and purple arrows. (A) All lacO and tetO arrays on Chr XIV were integrated between convergent genes and the terminators of the convergent genes were duplicated such that each copy flanked the insertion site. In this way all genes retained wild type copies of their terminators after insertion. (B) PolII enrichments at the integration sites of the 70.6kb strain. TetO array was integrated between YDL089w and YDL088c, and lacO array was integrated between YDL055c and YDL054c. (C) PolII enrichments at the integration sites of the 25.3kb strain. This strain is flanked by the ura3-1 point mutant and wild type URA3.

1. Mayer A, Heidemann M, Lidschreiber M, Schreieck A, Sun M, Hintermair C, Kremmer E, Eick D, Cramer P: CTD tyrosine phosphorylation impairs termination factor recruitment to RNA polymerase II. Science2012, 336(6089):1723-1725.
2. Weiner A, Hsieh TH, Appleboim A, Chen HV, Rahat A, Amit I, Rando OJ, Friedman N: High-resolution chromatin dynamics during a yeast stress response. Mol Cell 2015, 58(2):371-386.
3. Bonnet J, Wang CY, Baptista T, Vincent SD, Hsiao WC, Stierle M, Kao CF, Tora L, Devys D: The SAGA coactivator complex acts on the whole transcribed genome and is required for RNA polymerase II transcription. Genes Dev2014, 28(18):1999-2012.
4. Yen K, Vinayachandran V, Batta K, Koerber RT, Pugh BF: Genome-wide nucleosome specificity and directionality of chromatin remodelers. Cell 2012, 149(7):1461-1473.
5. Qian H, Sheetz MP, Elson EL: Single particle tracking. Analysis of diffusion and flow in two-dimensional systems. Biophys J1991, 60(4):910-921.

6. Mayer A, Lidschreiber M, Siebert M, Leike K, Soding J, Cramer P: Uniform transitions of the general RNA polymerase II transcription complex. Nat Struct Mol Biol 2010, 17(10):1272-1278.
