# Supplementary material for: High resolution imaging reveals heterogeneity in chromatin states between cells that is not inherited through cell division
Source: BMC Cell Biol. 2016 Sep 8;17(1):33. doi: 10.1186/s12860-016-0111-y (PMC5016949; doi:10.1186/s12860-016-0111-y)
Supplement: Additional file 2: — Plasmids and strains used in this study. (PDF 460 kb) [file 12860_2016_111_MOESM2_ESM.pdf]

Plasmid list		Origin
pAT253corrected	lacI**-GFP::HIS3	Dubarry, M
pT1196	pYiplac128TetRGFP::LEU2	Renshaw, M
pYCG_YLR106c	MDN1	EUROSCARF
pLAU43	lacO non-repeating	Lau, I
pLAU44	tetO non-repeating	Lau, I
pAG32	HygR	McCusker, J
pAG25	pFA6natMX4	McCusker, J
pAG25-3xGFP	3xGFP	Tanaka, T. U.
pKS391	mCherry::NAT	Snaith, H
pAFS135	lacI-GFP::HIS3	Straight, A
pAFS59	lacOx256	Straight, A
pRS306tetO224	TetOx224	Michaelis, C
pRS416	URA3	Sikorski, R
pFA6KanMX6	KanR	Wach, A
pDD2193	pAG25-TetR-3xGFP::NAT	This study
pDD202	tetO for MDN1	This study
pDD206	Gal1p-MDN1	This study
pDD207	MDN1-lacO	This study
pDD2244	tetR-GFP-TetR-mCherry::ADE2 cl.2	This study
pDD2245	GFP-lacI**-TetR-mCherry::ADE2 cl.1	This study
pDD2246	TetO-UBP10::TRP1 cl.5.1 for DD1407 and DD1473	This study
pDD2247	tetR-mCherry::NAT cl.3 for 1336	This study
pDD2248	tetR-mCherry::HygR cl.1 for 1413	This study
pDD249	42.3kb LacO-SSU72 cl.8	This study
pDD250	42.3kb TetO-ZWF1 cl.6	This study
pDD251	51.3kb LacO-INN1 cl.4 (and 91kb)	This study
pDD252	51.3kb TetO-SPC98 cl.1	This study
pDD2577	64.8kb TetO-UBP10 cl. 5.1	This Study
pDD253	64.8kb LacO-INN1 cl.1	This study
pDD254	71kb LacO-SSU72 cl.1	This study
pDD255	71kb TetO-UBP10 cl.1	This study
pDD256	100.8kb TetO-OCA1 cl.15	This study
pDD71/2580	LacO::URA3	This study
pDD72/2581	TetO::TRP1	This study

Yeast strains

Strain	Genotype	Origin	Total kb	Chr	tetO integration Start	tetO integration End	lacO integration Start	lacO integration End
DD1407	Mat**a** can1-100 his3-11,15 leu2-3,112 ura3-1 TetR-GFP-TetR-mCherry::ADE2 tetOx240::TRP1 at UBP10	This study	NA	XIV	UBP10	MRPL19	NA	NA
DD1413	Mata ade2-1 can1-100 leu2-3,112 GFP-lacI::HIS3 tetR-mCherry::HygR lacOx256-Gal1p-MDN1-tetOx224 at URA3 locus	This study	25.3	V	ura3			URA3
DD1471	Mata can1-100 his3-11,15 leu2-3,112 GFP-lacI**-tetR-mCherry::ADE2 tetOx240-ZWF1::TRP1 lacOx240-SSU72::URA3	This study	42.3	XIV	ATG2	ZWF1	SSU72	SNR19
DD1472	Mata can1-100 his3-11,15 leu2-3,112 GFP-lacI**-tetR-mCherry::ADE2 tetOx240-SPC98::TRP1 lacOx240-INN1::URA3	This study	51.3	XIV	INN1	RPC31	SPC98	ESBP6
DD1473	Mata can1-100 his3-11,15 leu2-3,112 GFP-lacI**-tetR-mCherry::ADE2 tetOx240-UBP10::TRP1 lacOx240-INN1::URA3	This study	64.8	XIV	UBP10	MRPL19	INN1	RPC31
DD1336	Mata ade2-1 can1-100 leu2-3,112 trp1-1 ura3-1 LacOx256 at 358kb tetOx224 at 298kb his3::GFP-lacI::HIS3 ade1::tetR-mCherry::Nat	This study	70.6	IV	NUR1	ASM4	PSA1	MCH1
DD1474	Mata can1-100 his3-11,15 leu2-3,112 GFP-lacI**-tetR-mCherry::ADE2 tetOx240-UBP10::TRP1 lacOx240-SSU72::URA3	This study	71	XIV	SSU72	SNR19	UBP10	MRPL19
DD1475	Mata can1-100 his3-11,15 leu2-3,112 GFP-lacI**-tetR-mCherry::ADE2 tetOx240-OCA1::TRP1 lacOx240-INN1::URA3	This study	100.8	XIV	INN1	RPC31	MIC27	OCA1
